# Importancia de la determinación de variantes en el número de copias en neonatos con aneuploidías autosómicas

**DOI:** 10.7705/biomedica.5354

**Published:** 2021-06-15

**Authors:** Hugo Abarca, Milana Trubnykova, Félix Chavesta, Marco Ordóñez, Evelina Rondón

**Affiliations:** 1 Servicio de Genética y EIM, Instituto Nacional de Salud del Niño-Breña, Lima, Perú Instituto Nacional de Salud del Niño-Breña Lima Perú; 2 Carrera Profesional de Medicina Humana, Universidad Científica del Sur, Lima, Perú Universidad Científica del Sur Universidad Científica del Sur Lima Peru; 3 Facultad de Medicina Humana, Universidad Ricardo Palma, Lima, Perú Universidad Ricardo Palma Universidad Ricardo Palma Lima Peru; 4 Escuela de Medicina Humana, Universidad Nacional de San Antonio Abad de Cusco, Cusco, Perú Universidad Nacional San Antonio Abad del Cusco Universidad Nacional de San Antonio Abad de Cusco Cusco Peru

**Keywords:** aneuploid**í**a, variaciones en el número de copia de ADN, recién nacido, trastorno del neurodesarrollo, sordera, Aneuploidy, DNA copy number variations, infant, newborn, neurodevelopmental disorders, deafness

## Abstract

**Introducción.:**

Las aneuploidías son trastornos genéticos frecuentes en la práctica clínica; sin embargo, se conoce poco sobre las otras variantes genéticas que modifican el fenotipo final.

**Objetivo.:**

Determinar las variantes en el número de copias y las regiones con pérdida de heterocigosidad autosómica mayor de 0,5 % o de regiones mayores de 10 Mb en neonatos con aneuploidías autosómicas*.*

**Materiales y métodos.:**

Se hizo el análisis cromosómico por micromatrices a los neonatos con aneuploidías autosómicas (n=7), trisomía 21 (n=5) y trisomía 18 (n=2) evaluados en los hospitales Antonio Lorena y Regional de Cusco, Perú, en el 2018.

**Resultados.:**

En dos neonatos se encontraron variantes en el número de copias, patogénicas o probablemente patogénicas, en regiones diferentes al cromosoma 21 o al 18. Además, se observaron dos variantes del número de copias con más de 500 kpb de patogenia desconocida.

**Conclusiones.:**

Si bien el número de pacientes era muy reducido, es importante resaltar que se encontraron otras variantes en el número de copias que se han descrito asociadas con trastornos del neurodesarrollo, varias anomalías congénitas, hipoacusia y talla baja o alta, entre otras, lo que probablemente influye negativamente en el fenotipo de este grupo de pacientes.

## Abstract (en)

**Introduction:**

Aneuploidies are frequent genetic disorders in clinical practice. However, little is known about other genetic variants that may influence the final phenotype.

**Objective:**

To determine the variations in the number of copies and regions with homozygosity greater than 0.5% or larger than 10 Mb in newborns with autosomal aneuploidies.

**Materials and methods:**

We performed a chromosomal microarray analysis on newborns with autosomal aneuploidies (n=7), trisomy 21 (n=5), and trisomy 18 (n=2) evaluated at the *Hospital Antonio Lorena* and *Hospital Regional of Cusco,* Perú, during 2018.

**Results:**

We found pathogenic and probably pathogenic variants in the number of copies in other genomic regions different to chromosomes 21 or 18 in two neonates. Additionally, we found two variants bigger than 500 kpb of unknown pathogenicity.

**Conclusions:**

Although the number of analyzed individuals was small, it is important to highlight that we found other variants in the number of copies that have been described in association with neurodevelopmental disorders, congenital anomalies, deafness, and short/ tall stature, among others, in almost half of them, which will probably impact the phenotype negatively in patients with aneuploidies.

Las aneuploidías autosómicas más frecuentes en recién nacidos son la trisomía 21 (síndrome de Down), la trisomía 18 (síndrome de Edwards) y la trisomía 13 (síndrome de Patau) ^1^. En Estados Unidos, se estima que uno de cada 732 recién nacidos vivos presenta el síndrome de Down, en tanto que la incidencia reportada del síndrome de Edwards es de uno de cada 4.857 a 10.000 y, la del síndrome de Patau, de 1,4 de cada 10.000 ^2^^-^^4^.

El análisis cromosómico por micromatrices permite detectar las variaciones en el número de copias (*Copy Number Variation,* CNV), las cuales se definen como segmentos de ADN iguales o mayores de 1 kpb; además, esta técnica permite describir regiones de homocigosidad (*Regions of Homozygosity,* ROH), cuyo índice se utiliza para evaluar disomías uniparentales y consanguinidad parental no declarada ^5^.

Las variaciones en el número de copias son frecuentes en el genoma humano e influyen en la variación fenotípica interindividual ^6^; pueden ser de pérdida o de ganancia y provocan un fenotipo determinado según el número de genes comprometidos o si son genes cuyo efecto depende del número de copias, la modificación transcripcional o la fusión génica ^7^^,^^8^.

Dichas variaciones resultan en fenotipos con discapacidad intelectual, retraso del desarrollo psicomotor, epilepsia, trastorno del espectro autista, esquizofrenia y anomalías congénitas ^9^^,^^10^. Por ejemplo, las variaciones de ganancia de los genes *BCAP31* y *SLC6A8* se asocian con hipoacusia no sindrómica ^11^^,^^12^ y, las de los genes *SHOX* y *PAK2*, con trastornos del espectro autista y del neurodesarrollo ^13^^-^^21^. También, se ha descrito la relación de las variaciones en el número de copias y las anomalías congénitas, como cuando hay compromiso del gen *TTC28* y se producen anomalías congénitas del tubo digestivo ^22^. Debe señalarse que algunas de estas variaciones se han descrito solamente a partir de bases de datos, como la DECIPHER (*Database of Chromosomal Imbalance*, *Phenotype in Humans using Ensemble Resources*) ^23^, y que las manifestaciones clínicas son pleiotrópicas, como la variación de ganancia en el cromosoma 16 (p12.2), la cual se ha relacionado con trombocitopenia, trastornos del neurodesarrollo y riñones en herradura ^23^^,^^24^.

También, se ha reportado que algunas variaciones frecuentes en el número de copias están asociadas con enfermedades comunes, como infección por HIV, glomerulonefritis, lupus eritematoso sistémico, enfermedad de Crohn, psoriasis, osteoporosis, neuroblastoma, talla baja, fisura labio- palatina y obesidad; en este mismo sentido, algunas variaciones raras se han relacionado con la enfermedad de Parkinson, la enfermedad de Alzheimer y el trastorno bipolar ^6^.

En el síndrome de Down, se observan características clínicas diversas, por ejemplo, discapacidad intelectual, cardiopatías congénitas (40 %), defectos gastrointestinales (~8 %), leucemia linfoblástica (~20 %) o trastorno mieloproliferativo transitorio neonatal (~10 %).

Se ha descrito que las variaciones frecuentes en el número de copias del mismo cromosoma 21 pueden determinar, por ejemplo, un mayor riesgo de enfermedad de Alzheimer, leucemia megacarioblástica aguda, hipoacusia o defectos congénitos ^6^. Es así que, en pacientes con trisomía 21, se ha observado que las variaciones de pérdida del cromosoma 21 (por ejemplo, chr21:43,193,374-43,198,244 y chr21:43,411,411-43,413,231) se asocian con un menor riesgo de aparición de cardiopatías congénitas que las de duplicación u otras variantes polimórficas (*Single Nucleotide Variation,* SNV) de genes como el *CRELD1* y el *GATA4,* que predisponen a un mayor riesgo de padecerlas ^25^^,^^26^.

La discapacidad intelectual en los niños con síndrome de Down no está determinada únicamente por el efecto del número de copias de algunos genes como el *DYRK1A*, pues también se ha observado que algunas variantes polimórficas de nucleótido único (SNV) influyen negativamente en su capacidad intelectual ^27^^,^^28^.

En Perú, y en muchos países latinoamericanos, el diagnóstico prenatal de aneuploidías no está protocolizado, y la legislación prohíbe la terminación de la gestación en el caso de anomalías congénitas ^29^. En tal sentido, en un reporte previo se observó una incidencia de neonatos con síndrome de Down hasta tres veces mayor que lo reportado mundialmente ^30^.

En este contexto, es importante determinar con mayor precisión cuáles son las variantes genéticas que podrían modificar negativamente el fenotipo de las aneuplodías (por ejemplo, de la trisomía 21), con el fin de conocer con exactitud el pronóstico y ofrecer tratamientos específicos.

## Materiales y métodos

El estudio fue descriptivo y transversal. Se preservó la privacidad y la confidencialidad de los datos clínicos y genómicos de los sujetos de investigación y sus respectivas familias, siguiendo las directrices de las buenas prácticas clínicas y de ética de la investigación biomédica. El estudio fue autorizado por el Comité de Ética Independiente del Instituto Nacional de Salud del Niño-Breña y se contó con la firma del consentimiento informado por parte de los padres.

Durante el periodo de estudio, nacieron en los hospitales Regional y Antonio Lorena de Cusco 19 niños con aneuploidías, 16 de ellos con trisomía 21 libre y tres con trisomía 18; no se registró ningún paciente con trisomía 13.

Se utilizó un cuestionario estructurado para la recopilación de la información clínica y molecular, y se codificaron y encriptaron los datos de identificación de los pacientes. El análisis cromosómico por micromatrices (*Chromosomal Microarray Analysis*, CMA) se hizo en una muestra de sangre periférica, de la cual se extrajo el ADN genómico (250 ng). El ADN fue amplificado, etiquetado e hibridado, usando el protocolo GeneChip CytoScan 750K Array™ (Affymetrix, USA) y siguiendo las instrucciones del fabricante.

La prueba incluye 550.000 marcadores no polimorfos y 200.436 marcadores de polimorfismo de nucleótido único (*Single Nucleotide Polymorphism*, SNP). Las celdas en filas se escanearon y luego se analizaron mediante el programa informático *Chromosome Analysis Suite* (ChAS)™ (Affymetrix, USA)*.* Las ganancias o pérdidas se consideraron si comprometían, por lo menos, 50 y 25 marcadores, respectivamente, y las regiones de homocigosidad, si abarcaban una longitud de por lo menos 5 Mb. Para determinar el número de recién nacidos vivos con aneuploidías autosómicas en los que se realizó el análisis cromosómico por micromatrices, se calculó el tamaño de la muestra según la fórmula (n = [N * (z^2^ * p * q)] / [e^2^ * (N - 1) + z^2^ * p * q), en la cual N=7.413, z=99 %, e=5 % y p correspondía a la incidencia de las aneuploidías 21 y 18, por lo que se determinó que n_1_=5 correspondía a la trisomía 21 y n_2_=2 a la trisomía 18. No se hizo el análisis cromosómico por micromatrices ni ningún estudio citogenético a los padres ni a la población en general.

Las variaciones en el número de copias se compararon con las bases de datos genómicos DECIPHER, y la de la *University of California*, Santa Cruz (UCSC), y se clasificaron en patogénicas, probablemente patogénicas, de significado incierto, probablemente benignas o benignas, según las recomendaciones del *American College of Medical Genetics and Genomic* (ACMG) ^31^^,^^32^.

Los resultados del análisis cromosómico por micromatrices se clasificaron como anormales si se demostraban variaciones en el número de copias patogénicas o probablemente patogénicas, o si se presentaba, por lo menos, una región con una región de homocigosidad mayor de 10 Mpb o si el total de las regiones de homocigosidad en las regiones autosómicas era mayor de 0,5 % ^33^.

Por último, se determinó la frecuencia de los resultados anormales del análisis cromosómico por micromatrices y se comparó con los estudios previos publicados. El análisis descriptivo se hizo mediante el uso de frecuencias y porcentajes.

## Resultados

Entre diciembre del 2017 y noviembre del 2018, hubo en el Hospital Antonio Lorena 3.195 recién nacidos vivos y, en el Hospital Regional de Cusco, 4.218 (N=7.413). El número de estos con síndrome de Down en las dos instituciones fue de 16, estimándose una incidencia de 2,16 por cada 1.000 recién nacidos vivos. Los antecedentes prenatales, el tipo de parto, las anomalías congénitas asociadas, las características antropométricas, la edad de los padres y la edad gestacional, se detallan en el cuadro 1. Tres de los recién nacidos presentaban síndrome de Edwards, es decir, una incidencia de 0,4 por cada 1.000 recién nacidos vivos. No se registró ningún paciente con síndrome de Patau.

**Cuadro 1:** Detalle de los antecedentes prenatales, tipo de parto, anomalías congénitas asociadas, características antropométricas, edad parental y edad gestacional

Recién nacidos con trisomía 21 (n=16)		
Sexo	n	%
Masculino	8	50
Femenino	8	50
Antecedentes prenatales	n	%
No	5	31,25
Consumo de alcohol	3	18,75
Fiebre en el primer trimestre	2	12,5
Amenaza de aborto	2	12,5
Amenaza de parto prematuro	2	12,5
Parto	n	%
Vaginal	7	43,75
Cesárea	9	56,25
Otras anomalías asociadas	n	%
Malformación anorrectal	2	12,5
Atresia esofágica	1	6,25
Malformación cardíaca	7	43,75
Antropometría al nacer	Mediana	Rango intercuartílico
Peso (g)	3.040	1.890-3.310
Talla (cm)	47.7	43-48
Perímetro cefálico (cm)	33	28-35
Edad parental	Mediana	Rango intercuartílico
Padre	32	20-46
Madre	29	19-42
Edad gestacional	Mediana	Rango intercuartílico
Total	38	31-39

La mediana del porcentaje de las regiones de homocigosidad fue de 0,47 %, con valores que fluctuaron entre 0,37 y 0,93 %. Tres de los neonatos tenían una regi**ó**n de homocigosidad mayor de 0,5 % y ninguno presentó una regi**ó**n de homocigosidad en un cromosoma mayor de 10 Mb que indicara una posible disomía uniparental (cuadro 2).

**Tabla 2:** Tamaño de las regiones de homocigosidad y coeficiente de endogamia en los recién nacidos vivos con aneuploidiías en Cusco

Parentesco	Grado de relación	Coeficiente de endogamia	Proporción teórica idéntica en descendientes	Rango de Mb	Promedio Mb	Rango de %	Número de RNV con ROH
Padres-hijos; hermanos	Primer	1/4	25 %	540 1.080	720	18,7 37,5	0
Tío-sobrino; primos de primer grado doble	Segundo	1/8	12,50 %	270 539	360	9,37 18,7	0
Primos de primer grado	Tercer	1/16	6,25 %	135 269	180	4,69 9,34	0
Primos de segundo grado	Cuarto	1/32	3,13 %	68 134	90	2,36 4,65	0
Primos de tercer grado	Quinto	1/64	1,56 %	14 67	43,75	0,5 2,35	3

RNV: recién nacidos vivos; ROH: *regions of homocigocity*

**Figura 1:**
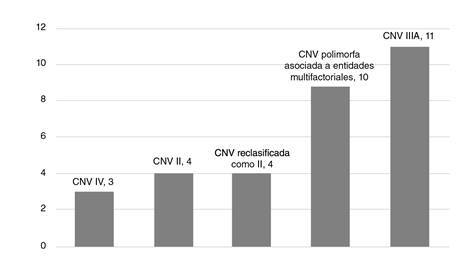
Proporción de variantes en el número de copias encontradas en recién nacidos con trisomía 21 y trisomía 18. Las variantes en el número de copias de significado incierto (tipo IIIA) fueron las más frecuentes, seguidas por las polimorfas, las IIIA reclasificadas como probablemente patogénicas (II), las II y, por último, las benignas (IV).

En total, hubo 34 variaciones en el número de copias, con un tamaño variable que osciló entre 28 y 77.878 kb y una mediana de 165 kb. En total, hubo 42 variaciones en el número de copias, con un tamaño variable que osciló entre 28 y 77.878 kb y una mediana de 165 kb entre variantes que van desde las patogénicas hasta las benignas, según la clasificación del ACMG. Las variantes patogénicas están relacionadas con la trisomía 18 y 21. Se encontraron variaciones benignas en tres y probablemente benignas en un neonato. Hubo 10 variaciones polimorfas en el número de copias que se han asociado a carcinoma nasofaríngeo en cuatro, a fisura labio-palatina en tres, a obesidad precoz en dos y talla baja en uno. De las variaciones de capacidad patógena desconocida, 10 tuvieron un tamaño menor de 500 kb. (figura 1).

En un recién nacido con trisomía 21, se encontraron otras tres variaciones en el número de copias probablemente patogénicas en 3q29, Xp22.33 y Xq28. Por otro lado, en una de las trisomías 18, se encontró una variación probablemente patogénica en 16p12.2 (cuadro 3).

**Cuadro 3:** Variantes en el número de copias patogénicas y probablemente patogénicas en recién nacidos con trisomía 21 y trisomía 18

Paciente	Región cromosómica	Tipo de CNV	Coordenadas	Tamaño (kb) Número de genes	Clase	Genes asociados a fenotipo	Dosis génica+	Identificación DECIPHER	Fenotipo esperado
1	21q	Duplicación	15,016,486_48,093,361	>33.000 511	I	DYRK1A1	0		Síndrome de Down
2	21q	Duplicación	15,016,486_48,093,361	>33.000 511	I	DYRK1A1	0		Síndrome de Down
3	16p12.2	Duplicación	21,740,199_22,442,007	702 18	II			294453; 304664; 305863	Riñones en herradura, trombocitopenia, TEA
	18p11.32q23	Duplicación	136,227-78,013,728	>77.000 697	I		0		
4	21q	Duplicación	15,016,486_48,093,361	>33.000 511	I	DYRK1A1	0		Síndrome de Down
5	18p11.32q23	Duplicación	136,227-78,013,728	>77.000 697	I				Síndrome de Edwards
6	3q29	Deleción	196,507,578-196,535,849	28 2	II	PAK2	1; 16,97 %	--	Esquizofrenia, TEA
	21q	Duplicación	15,016,486-48,093,361	>33.000	I	DYRK1A1	0		Síndrome de Down
	Xp22.33	Duplicación	535,572-644,544	109 1	II	SHOX	0	--	Talla alta o baja y TND
	Xq28	Duplicación	152,927,530-153,102,573	175 11	II	SLC6A8, BCAP31	0	--	Hipoacusia
7	21q	Duplicación	15,016,486_48,093,361	>33.000 511	I	DYRK1A1	0		Síndrome de Down

TEA: trastorno del espectro autista; CNV: copy number variation; TND: trastornos del neurodesarrollo

+ Según ClinGen y DECIPHER;

En dos neonatos, se hallaron, además, variaciones en el número de copias de ganancia mayores de 500 kb en los cromosomas 4 y 8, clasificadas como de patogenia incierta, según DECIPHER y la USCC. Asimismo, se encontraron dos variaciones en el número de copias de pérdida que, según DECIPHER, incluían genes con un coeficiente de haploinsuficiencia menor o igual a 10 % (*ZZZ3*, *TTC28*) (cuadro 4).

**Cuadro 4:** Variantes en el número de copias clasificadas como desconocidas que podrían ser reclasificadas como probablemente patogénicas

Cromosoma	Tamaño (kb)	Nomenclatura de CMA	Tipo de CNV	Número de genes	Genes OMIM	Haploinsuficiencia <10 % o triplosensibilidad	Función	Puntaje según CNV Pathogenicity Calculator	Anomalías asociadas
4q31.1	691	arr[hg19] 4q31.	Duplicación	12	*UCP1*	--	Respiración		Comunicación
		1q31.21(140,937,627-141,628,406)x3					termogénica de las		interauricular
							mitocondrias		
					*MAML3*	--	Coactivador		
							transcripcional de		
							NOTCH		
					*CLGN*	--	Espermatogénesis		
					*ELMOD2*	--	Proteína activadora		
							de GTP		
					*TBC1D9*	--	Actúa como		
							GTPasa activando		
							proteínas Rab		
8p21.3	1,285	arr[hg19] 8p21.3(19,181,933- 20,466,541)x3	Duplicación	14	*LPL*	--	Hidrólisis de triglicéridos y VLDL	0	No especificado
					*LZST1*	--	Crecimiento celular		
					*SLC18A1*	--	Transportador vesicular de		
							monoaminas		
					*INTS10*	--	Subunidad del complejo		
							integrador,		
							asociado a la		
							polimerasa ARN II		
					*ATP6V1B2*	--	Acidificación de compartimentos		
							intracelulares		
1p31.1	108	arr[hg19] 1p31.1(78,108,972-	Deleción	3	*ZZ3*	ZZZ3	Modificaciones	0	Malformación
		78,216,987)x1					postraducción en		anorrectal
							histonas		
22q12.2	324	arr[hg19] 22q12.1(28,751,664-	Deleción	3	*TTC28*	TTC28	Condensación de	0.3	Malformación
		29,076,146)x1					los microtúbulos de		anorrectal
							la zona media del		
							huso		

CNV: *Copy number variation*; CMA: *Chromosomal microarray analysis*

No se pudo hacer el análisis cromosómico por micromatrices ni otros estudios citogenéticos a los padres de estos pacientes para tener una idea del estado de patogenia y evaluar si eran heredadas o *de novo*. En el mismo sentido, al no contar con este análisis en la población general, no fue posible determinar cómo las aneuploidias podrían influir en el riesgo de aparición de variaciones en el número de copias modificadoras del fenotipo o de su frecuencia poblacional.

## Discusión

En un estudio previo en un hospital de Lima, se había estimado una incidencia del síndrome de Down de 5,7 por 1.000 recién nacidos vivos ^30^; esta cifra es mayor que la encontrada en el presente estudio (2,16 por 1.000), la cual corresponde a los casos de dos hospitales. Esta información debe confirmarse mediante vigilancia epidemiológica de todos los hospitales de la región y del país, para incorporarla de manera progresiva en el Estudio Colaborativo Latinoamericano de Malformaciones Congénitas (ECLAMC) y contar con una base de datos más exacta.

En un paciente con trisomía 21 se encontró una ganancia de 175 kpb en el cromosoma X(q28), que comprometía nueve genes, entre ellos el *BCAP31* y el *SLC6A8,* cuya duplicación se ha relacionado con hipoacusia no sindrómica ^12^. El gen *BCAP31* (*B Cell Receptor Associated Protein 31*) codifica una proteína chaperona que se expresa en el retículo endoplasmático, cuya función es exportar proteínas secretadas desde dicho retículo y reconocer proteínas mal plegadas que están dirigidas hacia la vía de degradación asociada con este; además, sirve como receptor de carga para la exportación de proteínas transmembrana. Las variaciones en este gen se han relacionado con una alteración neurológica grave caracterizada por hipoacusia, distonía e hipomielinización central (MIM # 300475) ^34^. Sin embargo, según ClinGen y DECIPHER, aún no se sabe si es un gen cuyo efecto depende del número de copias (*gene dosage*) presentes en el genoma.

En el gen *SLC6A8,* que es un transportador de creatina encargado de proveer de energía al músculo esquelético y cardíaco, se ha observado que las variaciones en el número de copias de ganancia están asociadas con hipoacusia ^11^. Este paciente se encontró una variación en el número de copias de ganancia en Xp22.38 que comprometía al gen *SHOX*, el cual es un factor de riesgo y de baja penetrancia relacionado con los trastornos del espectro autista y otros trastornos del neurodesarrollo como la discapacidad intelectual ^17^^,^^21^.

Además, se ha observado una deleción de 28 kb en el cromosoma 3 (q29) que compromete parcialmente al gen *PAK2* (p21, *Proteín-Activated Kinase2*), el cual es una cinasa de serina o treonina que interviene en la supervivencia y el crecimiento celular por medio del citoesqueleto. El coeficiente de haploinsuficiencia de este gen es de 16,29 %, según DECIPHER, y de 1, según ClinGen, y las deleciones en él se han asociado con trastornos del espectro autista y esquizofrenia ^13^^-^^16^^,^^18^^,^^19^. En ratones, se ha observado que dichas deleciones están involucradas en el desarrollo cerebral y la patogenia de dichos trastornos ^20^. Es así que este recién nacido con trisomía 21 tiene un gran riesgo de presentar trastornos del espectro autista, esquizofrenia, hipoacusia o una mayor discapacidad intelectual.

En otro paciente con trisomía 18, se observó una variación de ganancia de 702 kpb en el número de copias de 12 genes en el cromosoma 16 (p12.2), la cual, según DECIPHER, se ha reportada como probablemente patogénica en tres pacientes (identificados como 294453, 304664 y 305863) y se ha asociado con trombocitopenia, trastornos del espectro autista, discapacidad intelectual y riñones en herradura ^23^^,^^24^.

Por otra parte, hubo tres pacientes con trisomía 21 y variaciones adicionales en el número de copias mayores de 500 kb en 4q31.1 y 8p21.2, así como otras que involucraban genes con haploinsuficiencia menor de 10 (cuadro 4), las cuales podrían reclasificarse como probablemente patogénicas por el tamaño y el contenido génico. En ese sentido, el paciente con estas variaciones en el número de copias, presentaba una malformación anorrectal, y se ha descrito que las variaciones en el gen *TTC28* están relacionadas con anomalías congénitas del tubo digestivo ^22^.

Por último, dos pacientes con trisomía 21 y uno con trisomía 18, cuyos padres no declararon consanguinidad, tenían una región de homocigosidad en los cromosomas autosómicos de entre 0,5 y 2,35 %, que corresponde a un coeficiente de endogamia (F) de 1/64, lo que indica un grado de relación parental de quinto grado. Esta consanguinidad podría ser un factor de riesgo de enfermedades recesivas autosómicas o, incluso, ser un factor etiológico de la aneuploidia ^35^.

Al no contar con estudios genéticos de los padres, como el análisis cromosómico por micromatrices, no fue posible establecer si las variaciones incidentales en el número de copias eran heredadas o *de novo* y, tampoco, reclasificar aquellas de significado incierto. Sin embargo, las variaciones patogénicas o probablemente patogénicas están relacionadas con otras condiciones y pueden incrementar la gravedad de algunos síntomas de los pacientes con aneuploidias.

**Figura 2:**
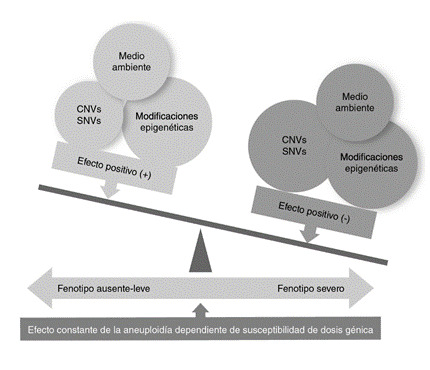
Interrelación de las variantes en el genoma de los niños con aneuploidías y el medio ambiente en la expresión final del fenotipo. Teóricamente las aneuploidías autosómicas provocan un fenotipo de manera constante en todos los pacientes (azul). Sin embargo, variantes genéticas diferentes a las aneuploidias ( o ) [variaciones en el número de copias (CNV) o las de nucleótido único (SNV)], conjuntamente con las modificaciones epigenéticas y factores del medio ambiente (por ejemplo, estimulación temprana deficiente) provocan un efecto negativo (rojo) en el fenotipo final (por ejemplo, mayor discapacidad intelectual, aparición de otros trastornos del neurodesarrollo).

Por lo tanto, el fenotipo variable que observamos en los pacientes con aneuploidias se debería a las diferentes variantes observables en el genoma en combinación y, aunque en menor proporción, al medio ambiente y los cambios epigenéticos (figura 2).

Las aneuploidias en los recién nacidos son el grupo de enfermedades genéticas más frecuentes, por lo que es de suma importancia conocer y reconocer cuáles son los factores genéticos o ambientales que modifican el fenotipo de las personas con aneuploidias como el síndrome de Down, para poder advertir oportunamente las complicaciones o el pronóstico y ofrecerles una atención adecuada.

Algunos de los recién nacidos en esta cohorte pequeña, tenían otras variaciones en el número de copias, las cuales modificarían el fenotipo de forma negativa y los haría más propensos a sufrir o que se agraven algunas de las características clínicas propias de las aneuploidías, por ejemplo, la hipoacusia en el síndrome de Down, o a padecer otras comorbilidades no descritas o muy poco frecuentes, como la esquizofrenia, la talla alta o baja y los trastornos del espectro autista, sobre todo aquellos con trisomía 21. Muchas veces, el fenotipo observado en estos pacientes es producto,o no solo de la aneuploidía observada *per se*, sino de otras variantes genéticas (variaciones en el número de copias o las de nucleótido único), las cuales deberían explorarse con más precisión y a mayor escala.
